# DNA damage and cell death induced by exposure to ultra-high dose rate low-dose pulsed X-rays emitted from a kilojoule plasma focus device

**DOI:** 10.1186/s40659-026-00674-1

**Published:** 2026-03-10

**Authors:** Héctor Araya, Jalaj Jain, Rodrigo Andaur, José Moreno, Sergio Davis, Pablo Diaz, Ethel Velásquez, Josefa Orellana, Martín Ríos, Jessica Toro, Octavio Orellana-Serradell, Cristopher Fierro, Leopoldo Soto, Katherine Marcelain

**Affiliations:** 1 Comisión Chilena de Energía Nuclear, Center for Research and Applications on the Intersection of Plasma Physics, Matter and Complexity (P2mc), Nueva Bilbao 12501, Las Condes, Santiago, Chile; 2 Departamento de Oncología Básico Clínica, Facultad de Medicina, Universidad de Chile, Avenida Independencia 1027, Santiago, 8380453 Chile; 3 Laboratorio de Biología Celular y Molecular, Departamento de Ciencias Básicas, Universidad Santo Tomás, Avenida Ejercito 146, Santiago, Chile; 4 Departamento de Ciencias Físicas, Universidad Andrés Bello, Republica 220, Santiago, Chile; 5 Escuela de Tecnología Médica, Facultad de Medicina – Clínica Alemana, Universidad del Desarrollo, Avenida Plaza 680, Las Condes, Santiago, Chile; 6 Departamento Vigilancia Sanitaria e Investigación, Subdepartamento de Investigación e Innovación, Instituto de Salud Pública de Chile, Avenida Marathon 1000, Ñuñoa, Santiago, Chile; 7 Centro de Investigaciones Nucleares para Aplicaciones en Salud y Biomedicina, Comisión Chilena de Energía Nuclear, Nueva Bilbao 12501, Las Condes, Santiago, Chile; 8 Centro para la Prevención y el Control del Cáncer, CECAN, Universidad de Chile, Av. Las Palmeras 299, Parque Quinta Normal, Santiago, Chile; 9 Facultad de Medicina Veterinaria y Agronomía, Instituto de Ciencias Naturales, Universidad de Las Américas, Sede Santiago, Campus Melipilla, Av. Jose Massoud Sarquis 482, Melipilla, Chile

**Keywords:** FLASH radiation, Cancer, Colorectal cancer, DNA damage, Cell death, Plasma focus device

## Abstract

**Background:**

FLASH radiotherapy, characterized by ultra-high dose rates (> 40 Gy/s), potentially spares normal tissues while maintaining antitumor efficacy (the FLASH effect). While electron and proton FLASH are explored, pulsed X-ray sources like plasma focus devices offer unique possibilities. Previous work has reported hyper-radiosensitivity in colorectal cancer cells exposed to ultra-high-dose-rate pulsed X-rays from a kilojoule plasma focus (PF) device, without significant effects on non-cancerous cells. This study further investigates the biological effects of ultra-high-dose-rate (~ 10⁷ Gy/min), low-total-dose pulsed X-rays generated by a PF-2 kJ device on colorectal cancer cell lines, focusing on DNA damage, cell cycle progression, and gene expression.

**Results:**

Low-total-dose (~ 0.25 Gy), ultra-high-dose-rate pulsed X-rays (0.025 Gy/pulse, a total of 10 pulses, pulses temporally separated by 15–20 s) generated by a PF-2 kJ device induced a significant increase in the SubG_1_ population in HCT116 and DLD1 cells over 72 h, an effect indicative of apoptosis, which was not observed with conventional X-rays at similar total doses. In addition, pulsed X-rays induced apoptosis in radioresistant MCF-7 breast cancer cells. Whereas conventional X-rays did not cause a significant increase in double-strand breaks (DSBs), surrogate marker γ-H2AX and phosphor-P53(Ser15) signal were detected 30 min following pulsed X-ray exposure and persisted for up to 24 h, and no evidence of G2/M cell cycle arrest was detected in exposed cells. Gene expression analysis and preliminary transcriptomic data further suggest a DNA damage response leading to cell death and global change in general biological processes related to regulation of gene expression.

**Conclusion:**

Low-total-dose (~ 0.25 Gy), ultra-high-dose-rate pulsed X-rays generated by a PF-2 kJ device induce significant and sustained DNA damage (DSBs) leading to increased apoptosis in colorectal (HCT-116, DLD-1) and breast (MCF-7) cancer cells, compared to conventional X-rays. These effects, coupled with distinct changes in gene expression, suggest that ultra-high-dose-rate pulsed X-rays may overcomeradio-resistancee without eliciting a conventional DNA damage repair or cell cycle checkpoint response. These findings support the potential of PF-generated pulsed X-rays as a novel sourcof e radiotherapy modality and warrant further investigation, particularly in in vivo models, to assess clinical applicability and safety.

**Supplementary Information:**

The online version contains supplementary material available at 10.1186/s40659-026-00674-1.

## Background

FLASH radiation involves the ultra-rapid delivery of radiotherapeutic doses at higher dose rates (~ 10^3^− 10^9^ Gy/s) than those from conventional radiation sources (< 40 Gy/a s or few Gy/s) [1–3]. Surprisingly, this short exposure time has shown relative protection of several normal tissues after a single dose, an effect that has been the called FLASH effect [2, 4–9]. This effect has been validated and reproduced in several animal models and a significant number of organs, including the skin, lungs, intestine, and brain [1, 10–12]. Thus, the FLASH effect is defined by its non-toxic effects on normal tissues, which would indicate a unique potential for its use in the clinic, for example, as an antitumor therapy. Despite its promise, studies of the FLASH effect in cancer are relatively limited, making this area a fertile ground for exploration given its distinctive characteristics and potential applications. In this regard, critical studies have been conducted in preclinical models of lung, ovarian, skin, intestinal, and brain cancer, wiindicatingts indicate that FLASH radiation was more effective in reducing tumor size and had fewer cytotoxic effects on normal tissues compared to conventional radiation [1, 3, 4, 7, 10, 13–17]. ​​ However, its specific clinical use remains undefined.

Current FLASH radiotherapy primarily employs electron pulses delivered in the microsecond range [1, 17]. Due to the superior depth penetration of protons, proton FLASH has also been explored, although studies in this area are still limited [3, 18]. In the last years, X-rays sources have re-emerged as an interesting radiation source to investigate the effects of nanosecond of radiation pulses on cancer cells [4, 6, 8, 9, 12, 19–22]. For instance, Litvyakov et al.irradiated mastocytoma P815, Ehrlich carcinoma, and K-562 erythromyeloleukemia cells with periodic X-ray pulses (exposure time 5 min at pulse repetition frequency of 3–40 Hz) at doses ranging from 30 to 120 mGy. They observed apoptosis in 20–40% of the cells and proposed that different apoptotic pathways might be activated by pulsed X-ray irradiation [23]. Shinohara et al. irradiated cultured L5178Y mouse cells and its radiosensitive XRCC4-deficient M10 cells using pulsed X-rays of sub-picoseconds emitted from laser-produced plasma at ultra-high dose rate of 10^12^–10^13^ Gy/s. Their work suggested that spatially dense ionization-induced cell death might differ from temporally dense ionization-induced cell death, and that the former may not increase the frequency of DNA double-strand breaks (DSBs) repaired by the Non-Homologous-End-Joining (NHEJ) mechanism [24]. Jain et al. demonstrated an increase in DNA double-strand breaks in the colorectal cell line DLD-1 at 30 min post-X-ray irradiation from a 100-joule plasma focus device 50 X-ray pulses, 0.12 Gy total dose, ~ 10 ns pulse duration measured using BPX65 PIN-diodes, ~ 2.4$\:\times\:$10^5^ Gy/sec dose rate, 0.0024 Gy/pulse [19]. The same group reported greater cell death in the colorectal cancer cell lines DLD-1, HCT-116, as well as the breast cancer cell line MCF-7, at significantly lower pulsed X-ray doses obtained from a kilojoule plasma focus device (~ 0.024 Gy per pulse, ~10^5^ Gy/s, a total of 10 X-ray pulses, and a total dose of ~ 0.25 Gy). Interestingly, the colorectal cancer cell line HCT-116 exhibits a hyper-radio-sensitivity phenomenon in response to pulsed X-ray irradiation, an effect not observed with conventional X-ray irradiation [20, 25]. Furthermore, a non-cancerous colorectal cell line, CCD-841-CoN, irradiated at the same dose that induced low-dose hyper-radiosensitivity in HCT-116 and DLD-1 cells, did not show significant death [20]. This highlights a key advantage in using this type of radiation in radioresistant tumor cells while protecting normal cells. Nevertheless, beyond experimental results, a few theoretical models have been proposed to describe biological outcomes of rapid dose deposition [5].

Plasma focus devices consist of a co-axial electrode geometry. The central electrode is normally considered an anode, which is partially covered by an insulator. The uncovered length of the anode is known as the anode effective length. At first, a plasma current sheet forms over the insulator and then runs along the anode effective length, where it compresses and forms a plasma column, known as pinch, at the anode top. At the time of compression and pinch charged particles (electrons, and ions) accelerate. Electrons move toward the anode bottom and produce X-rays via bremsstrahlung upon impinging on the anode bottom. Ions move away from the anode top and produce neutrons mainly via the beam-target nuclear fusion mechanism when deuterium is used as the working gas. Depending on the operating energy of the device that can range from 0.1 J to MJ, the time duration of the X-rays and neutron pulses can range from ns to hundreds of ns, being of the order of 100 ns for plasma focus devices operating at few kJ [26]. Additionally, metallic jets and axial plasma shocks are also observed, suggesting a great versatility of plasma focus devices for fundamental studies and their applications in varied scientific fields, as material sciences, biological sciences, nanotechnologies [27, 28].

In this study, we aimed to further characterize the biological effects of ultra-high-dose-rate (UHDR) pulsed X-rays (10 X-ray pulses at ~ 0.025 Gy per pulse dose, ~10^7^ Gy/min) by employing a PF-2 kJ plasma focus device. We investigated the induction of DNA double-strand breaks, changes in cell cycle distribution, apoptosis, and transcriptional changes in colorectal cancer cell lines as a research model.

## Materials and methods

### Cell culture

Human cell lines derived from colorectal cancer (HCT-116 and DLD-1) and breast cancer (MCF7) were obtained from American Type Culture Collection (ATCC, Rockville, MD, USA) and cultured with RPMI 1640 o DMEM culture media (Gibco, Life Technologies, NY, USA) supplemented with heat-inactivated fetal bovine serum (Mediatech), 100U/mL penicillin, 100 mg/mL streptomycin (Gibco, Life Technologies, NY, USA), as previously described [20, 29]. Cell cultures were incubated at 37 °C in a 5% CO_2_ atmosphere. The growth media were changed every 2 days. For pulsed X-rays experiments, cells were seeded in 4-chamber 35 mm dish (IBIDI, µ-Dish 35 mm Quad, Cat.No:80416, Germany) at a density of 8 × 10^4^ cells/well a day before irradiation. For experiments using continuous X-rays, 9.6$\:\times\:$10^4^ cells were seeded in 35 mm dish. For each experiment, control group cells were “mock-irradiated”.

## Cell irradiation

HCT-116, DLD-1 and MCF-7 cells were irradiated with 10 X-ray pulses (~ 0.25 Gy), using a kilojoule plasma focus device, PF-2 kJ, which served as a ultra-high dose rate (~ 0.025 Gy per pulse, ~10^7^ Gy/min, and a total dose of ~ 0.25 Gy) source [20]. Table 1 shows the dose and X-ray parameters. For comparison with conventional X-ray irradiation, a linear accelerator device (X1870940, Philips, Netherlands) was used to irradiate cell cultures at a dose of 0.3 Gy, with a dose rate of 0.5 Gy/min (10 mA, 180 kV, 4 mm Cu filter).

**Table 1:** Characteristics of X-ray pulses generated by the PF-2kJ plasma focus device

Dose/pulse (Gy)	Pulse duration at FWHM (ns)	Dose rate (Gy/s)	Dose rate (Gy/min)	Total dose (Gy)
~ 0.025	~ 90	~ 2.7$\:\times\:$10^5^	~ 1.7$\:\times\:$10^7^	0.25

TLD-100 dosimeters have been used to monitor doses for 10, 20, 30, and 40 X-ray pulses, and three independent dose measurements were carried out. Supplementary Fig. S1 shows the variation in doses with the number of X-ray pulses. Besides, doses were monitored before and after cell culture irradiation to ensure the dose repetition for 10 X-ray pulses.

## Indirect immunofluorescence

0.5, 3, 6 and 24 h post irradiation, cells were fixed using 4% p-formaldehyde for 10 min, permeabilized with 0.25% Triton X-100 at room temperature and blocked with 3% BSA/PBS for 45 min. Cells were incubated overnight with primary antibodies: anti-γ-H2AX (1:1000, Millipore, Temecula, CA, 05-636), anti-p53 (1:500, Cell Signaling, cat: 2524) and anti-Phospho-p53(1:500, Cell Signaling, cat: 9284) in 0.05% Tween-20 and 3% BSA/PBS. After washing, cells were incubated with anti-mouseb Alexa Fluor-488 secondary antibody (1:5000, Molecular Probes, A-21042) or anti-rabbit (1:5000, Invitrogen, cat: A11008). Slides were mounted with ProLong Gold Antifade Reagent with DAPI (Life Technologies, NY) and cells were photographed under a fluorescence microscope (BX53; Olympus, Japan). For γ-H2AX quantification, foci were counted using a Find Maxima plugin and normalized by number of nuclei using ImageJ software (Rasband, National Institutes of Health, USA).

## Real-time quantitative PCR

Total RNA was extracted from HCT-116 cell culture using Trizol Reagent (Invitrogen, 15596018) according to the manufacturer’s instructions and then quantified using a Nanodrop spectrophotometer (Cytation 3™, BioTek instruments). Subsequently, RNA was treated with RQ1 RNase-Free DNase (Promega, cat: M6101) for 30 minutes at 37°C to remove genomic DNA contamination. cDNA was synthetized using Affinity Script qPCR (Agilent Technologies, Santa Clara, CA, USA) and quantified using Brillant II Sybr (Agilent). RT PCR reaction was carried on in an Eco™ Real-time PCR System (Illumina Inc., San Diego, CA, USA. The relative expression levels of the target’ mRNAs were calculated by applying the 2^−∆∆CT^ method. HPRT-1 was used as housekeeping gene. The list of the used primers is in Table 2.

**Table 2:** List of primers used in RT-qPCR

Target	Primer (5’ to 3’)	Sequence
p21	Forward	CAGCAGAGGAAGACCATGTGGA
	Reverse	TTCCTGTGGGCGGATTAGGG
BRCA1	Forward	CTGAAGACTGCTCAGGGCTATC
	Reverse	AGGGTAGCTGTTAGAAGGCTGG
NOXA	Forward	TTGCGATTGGGATGCAGCTG
	Reverse	GATGCAGTCAGGTTCCTGAGCA
TP53	Forward	GTTCCGAGAGCTGAATGAGG
	Reverse	TTATGGCGGGAGGTAGACTG
Bcl-w	Forward	GGCAGCTGGAGATGAGTTCGA
	Reverse	GTCCCACCAGTGGTTCCATCT
HPRT-1	Forward	TGCTTTCCTTGGTCAGGCAGTA
	Reverse	CAACACTTCGTGGGGTCCTTTT
ZMAT3	Forward	ACCACAAAGCAGGGCAAGTT
	Reverse	AATGTTGGAGCTGGCGAAGA

## Flow cytometry and cell cycle analysis

A day before irradiation, 5 × 10^4^ cells/well of Colorectal cancer cells were seeded in 4-chamber 35 mm dish (IBIDI, µ-Dish 35 mm Quad, Cat.No:80416, Germany). After irradiation, cells were collected by trypzinization at 0, 24, 48 and 72 h, and then fixed with 70% ethanol at 4° C for 24 h. Following fixation, cells were stained with a DNA staining solution (50 µg/mL of Propidium Iodide, Sodium citrate 1% (w/v), 1% NP-40, 10 µg/mL of Rnase A and PBS 1X). Cell cycle distribution was evaluated by fluorescence-activated cell sorter FACSCanto (BD) and data was analyzed using FlowJo software (Treestar, Inc., San Carlos, CA).

### Trypan blue exclusion assay

Viability was evaluated by mixing cells with an equal volume of 0.4% trypan blue solution (Logos Biosystems, Gyunggi-Do, Korea). Cells were counted using a LUNA™ Automated Cell Counter (Logos Biosystem).

## RNA-sequencing (RNA-seq)

A total of 5 × 10^4^ HCT-116 cells were seeded in a 30 mm chamber dish and, 24 h later, exposed to 10 X-ray pulses (~ 0.25 Gy), 0.3 Gy conventional X-rays, or mock-exposed as control for each condition. One day after irradiations, the dishes were washed twice with 1X PBS and total RNA was extracted using the GenElute mRNA Miniprep Kit (Sigma-Aldrich, St. Louis, MO, USA). Only RNA samples with RIN ≥ 7 were selected for RNA-seq and sent to NovogeneTM for analysis. Messenger RNA was purified from total RNA using poly-T oligo-attached magnetic beads. After fragmentation, the first strand cDNA was synthesized using random hexamer primers, followed by the second strand cDNA synthesis using dUTP for directional library. Quantified libraries were pooled and sequenced in a Novaseq Illumina platform. An average of about 7.5G bases and 35 millions reads per sample were left after QC cleaning (> 92% QC30). No biological replicates were available for the analysis (*n* = 1). Before differential gene expression analysis, for each sequenced library, the read counts were adjusted by edgeR program package through one scaling normalization factor. The P values were adjusted using the Benjamini & Hochberg method. Corrected P-value of 0.05 and absolute foldchange of 2 were set as the threshold for significantly differential expression.

### Statistical analysis

The data were represented as mean ± S.D. from experiments with of three independent samples that were analyzed using one-way ANOVA with Tukey post-hoc tests to cytotoxic assay, or by using the Student’s *t*-test for flow cytometry and double strand break assay. *p*-values of *P* < 0.05 as statistically significant.

## Results

**Fig. 1:**
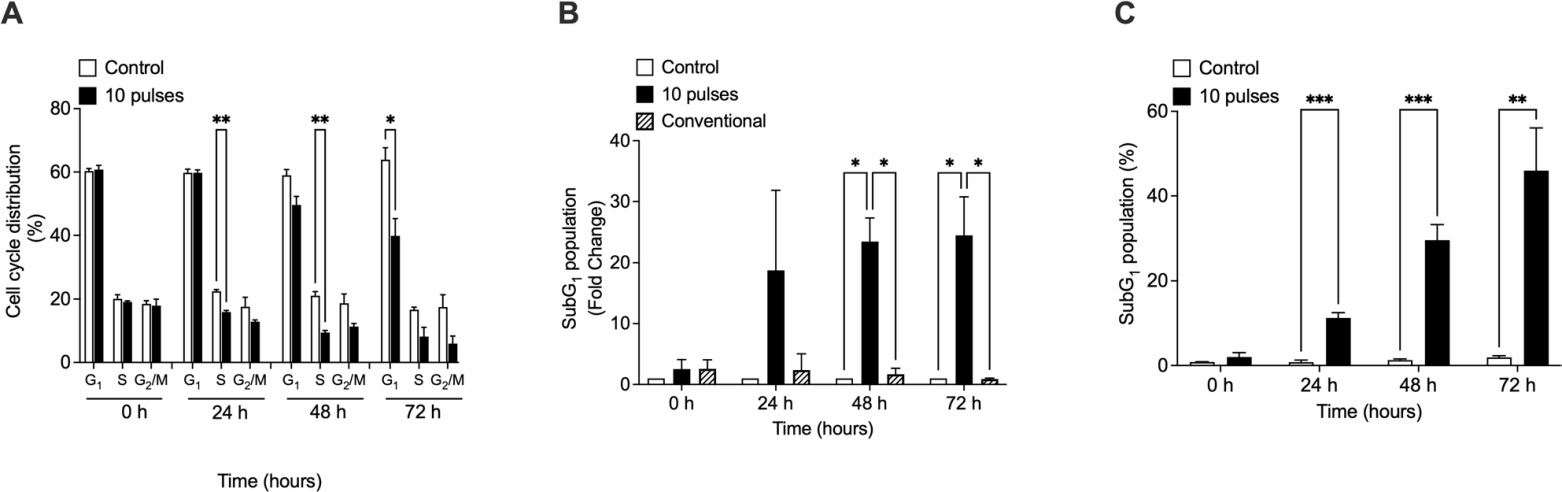
Pulsed x-rays from the PF-2Kj source induced apoptosis in HCT-116 cells. Colorectal derived cells lines were exposed to 10 pulses (0.25 Gy) and cell cycle distribution were evaluated at 0, 24, 48 and 72 h post irradiation by flow cytometry analysis assay (**A**). Apoptosis-induced was detected by SubG_1_ population quantification (**B**). Induction of apoptosis by pulsed and conventional x-rays (0.3 Gy) was compared with respect to not irradiated cells (control) at each time and expressed as Fold Change (**C**). Data represent three independent experiments for each condition. Results are shown as Mean ± S.D. *p < 0. 05; **p < 0.01; ***p < 0.001 respect to control cells for each time, using t-tests (**A** and** B**) or ANOVA with Tukey post hoc test (**C**)

To study the cytotoxic effects of pulsed X-rays, colorectal cancer cells, HCT-116, were exposed to 10 pulses of X-rays (~ 0.25 Gy), and cell cycle distribution was evaluated by flow cytometry at 0, 24, 48, and 72 h post-irradiation (Fig. 1A). The results showed that pulsed X-ray irradiation at 0.25 Gy evokes a reduction of ~ 10% of the cell population in the S stage, at 24 and 48 h post-irradiation, and 24% in the G_1_ stage at 72 h. The most notable effect was observed in the SubG_1_ population, which represents cells in apoptosis. As shown in Fig. 1B and C, pulsed X-ray irradiation induced a significant increase in apoptosis starting at 24 h and rising till 72 h post-irradiation. In contrast, cells exposed to conventional X-rays (0.3 Gy) exhibited no changes in the SubG_1_ population (Fig. 1C).

**Fig. 2:**
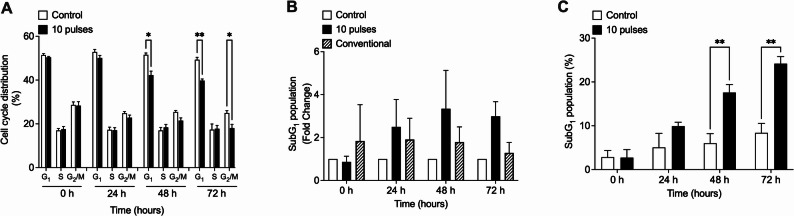
Pulsed x-rays from the PF-2Kj source induced apoptosis in DLD-1 cells. Colorectal derived cells lines were exposed to 10 pulses (0.25 Gy) and cell cycle distribution were evaluated at 0, 24, 48 and 72 h post irradiation by flow cytometry analysis assay (**A**). Apoptosis-induced in DLD-1 cells was detected by SubG_1_ population quantification (**B**). Induction of apoptosis in DLD-1 cells by pulsed and conventional x-rays (0.3 Gy) was compared with respect to nonirradiated cells (control) at each time and expressed as Fold Change (**C**). Data represent three independent experiments for each condition. Results are shown as Mean ± S.D. *p < 0.05; **p < 0.01; ***p < 0.001 respect to control cells for each time, using t-tests (Figure A and B) or ANOVA with Tukey post hoc test (**C**)

In the same way, pulsed X-ray irradiation alters cell cycle distribution in DLD-1 cells (Fig. 2A) and induces an increase in the SubG_1_ apoptotic fraction over time (Fig. 2B, C).

**Fig. 3:**
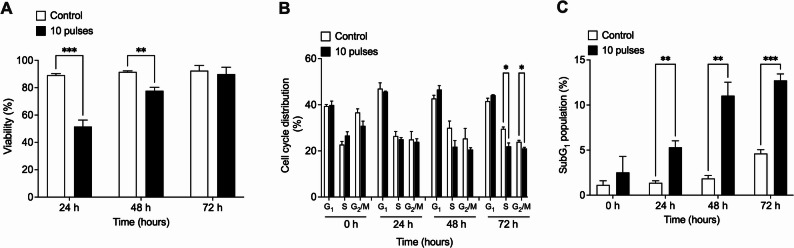
Pulsed x-rays from the PF-2Kj source induced apoptosis in radioresistant breast cancer cells lines, MCF-7. Cells were exposed to 10 pulses (0.25 Gy) and cell death was evaluated by trypan blue exclusion assay at 24, 48 and 72 h after irradiation. Data represent percentage of viability in each condition (**A**). Cell cycle distribution were evaluated at 0, 24, 48 and 72 h post irradiation by flow cytometry analysis assay (**B**). Apoptosis-induced was detected by SubG_1_ population quantification (**C**). Data represent three independent experiments for each condition. Results are shown as Mean ± S.D. *p < 0.05; **p < 0.01; ***p < 0.001 respect to control cells for each time, using t-tests

In a recent study, it was found that the DLD-1 cell line shows about 30% cell death when irradiated by conventional X-ray at 12 Gy of dose [29]. Here, about 25% of the DLD-1 cells have a SubG_1_ DNA content when irradiated by pulsed X-ray at 0.25 Gy of dose, which suggest a switch in the response of DLD-1 cells fromradio-resistancee to radio-sensitive to low doses. We explored whether this effect was also observed in a different radioresistant cell line [30]. MCF-7 cells exposed to 10 X-ray pulses show a reduction in viability in ~ 38%, 24 h post exposition (Fig. 3A), a slight decrease in S and G_2_/M population at 72 h post irradiation (Fig. 3B), and a significant increased the SubG_1_ population (Fig. 3C). These effects are not observed with low doses conventional X-rays [31, 32].

**Fig. 4:**
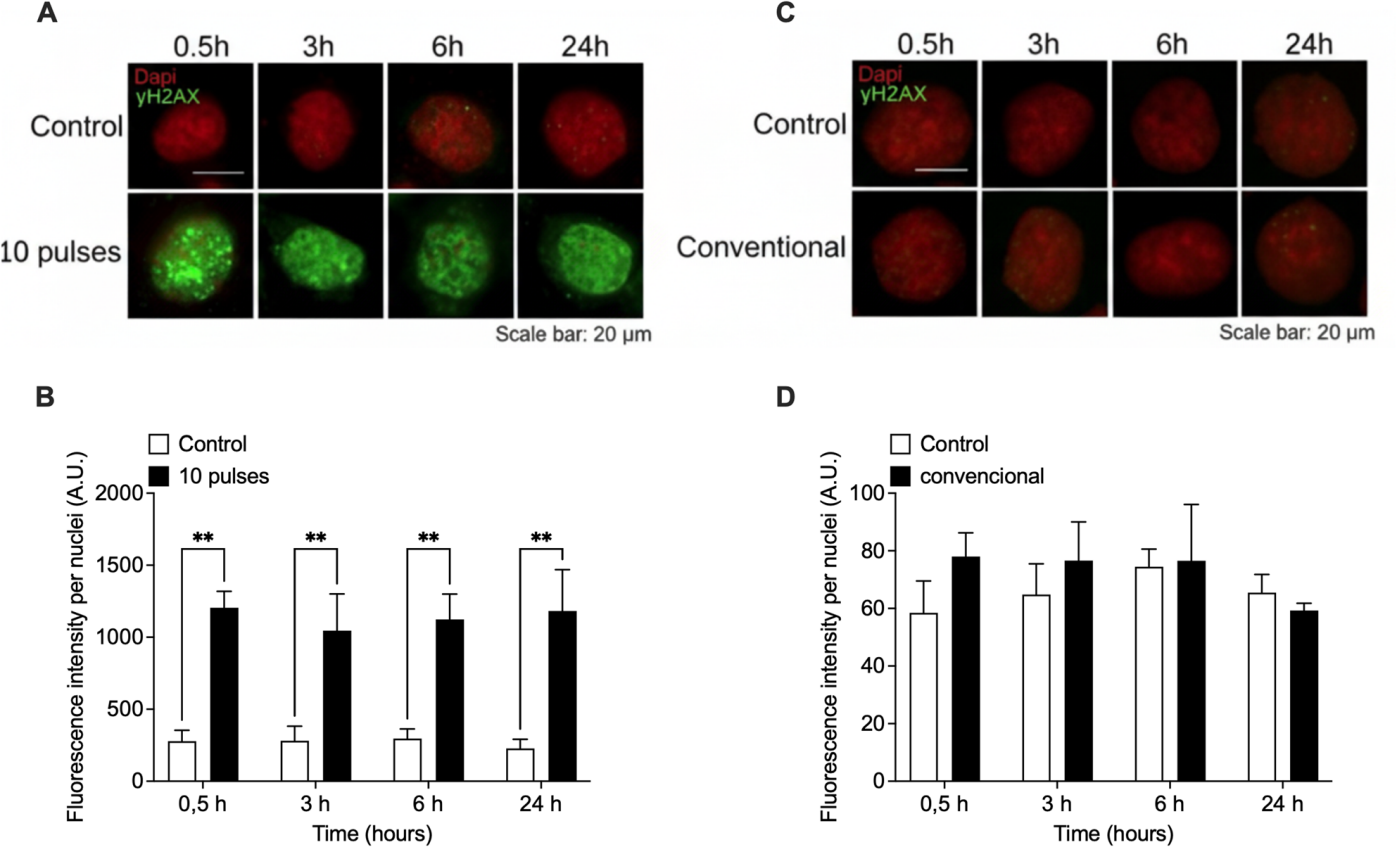
Pulses radiation induce DSBs in HCT-116 cells. Evaluation of double strand break (DSB) marker $\:\gamma\:$-H2AX by immunofluorescence at several times after exposure to 10 pulses (**A**) or 0.3 Gy of conventional x-rays (**C**). Nuclei were stained with DAPI (red) and intensity of $\:\gamma\:$-H2AX signal (green) was quantified and normalized to the number of nuclei (**B** and** D**). Signal was evaluated from 5 different fields and at least 100 cells were evaluated per sample. Results are shown as Mean ± S.D. of 3 independent experiments. **P < 0.01 respect to control cells for each time, using t-tests

**Fig. 5:**
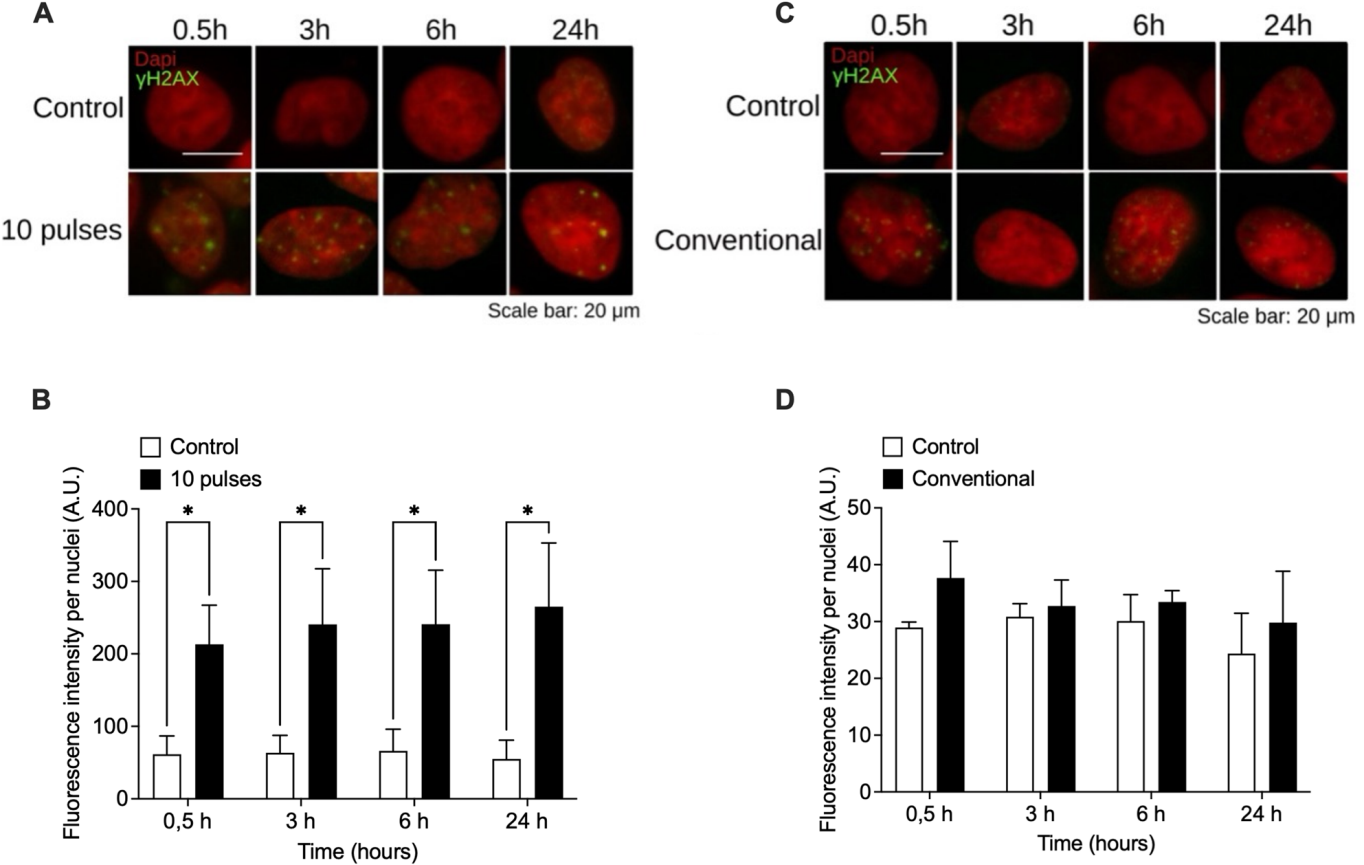
Pulses radiation induces DSBs in DLD-1 cells. Evaluation of double-strand break (DSB) marker γ-H2Ax by immunofluorescence at several times after exposition to 10 pulses (**A**) or 0.3 Gy of conventional x-rays (**C**). Nuclei were stained with DAPI (red) and intensity of γ-H2Ax signal (green) was quantified and normalized to the number of nuclei (**B** and **D**). Signal was evaluated from 5 different fields and at least 100 cells were evaluated per sample. Results are shown as Mean ± S.D. of 3 independent experiments. **P < 0.01 respect to control cells for each time, using t-tests

To assess the underlying mechanisms of pulsed X-ray-induced cell death, we evaluated the generation of DNA double-strand breaks (DSBs), by detecting the surrogate biomarker, γ-H2AX. HCT116 and DLD-1 cells were exposed to 10 pulses of X-rays (0.25 Gy) or conventional radiation (0.3 Gy) and phosphorylation of H2AX (visualized as γ-H2AX foci) was evaluated by immunofluorescence at 0.5, 3, 6 and 24 h post irradiation (Figs. 4 and 5). Pulsed X-rays induced a significant increase in DSBs 30 min post-irradiation. On the contrary, conventional X-ray irradiation did not induce significant DNA DSBs. Interestingly, DSBs induced by pulsed X-rays seems not to be repaired, since γ-H2AX signal remains unchanged 24 h post-irradiation.

These findings indicate that pulsed X-ray of low-energy (8–15 keV) with ultra-high-dose rate and a total low dose (~ 0,25 Gy) can cause DNA damage and promote apoptotic cell death, with a relative biological effectiveness like high-LET radiations.

**Fig. 6:**
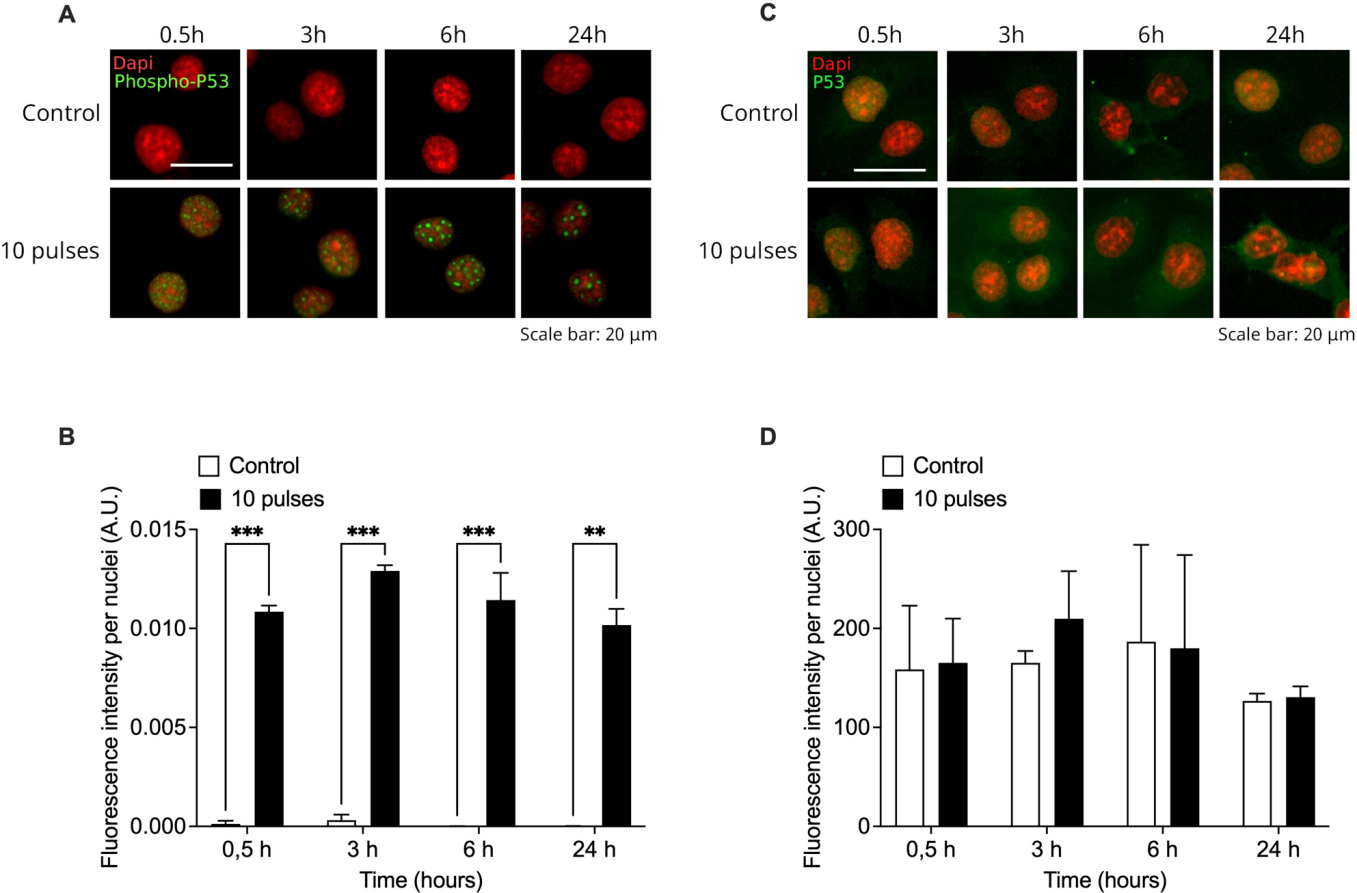
Radiation pulses induce phosphorylation of the DNA damage response protein p53. Levels of phospho P53 (**A**) and total P53 (**C**) were evaluated by immunofluorescence in HCT-116 cells at several times after exposure to 10 pulses. Nuclei were stained with DAPI (red) and intensity of phospho P53 and P53 signal were quantified and normalized to the number of nuclei (**B** and **D**). Signal was evaluated from 3 different fields and at least 100 cells were evaluated per sample. Results are shown as Mean ± S.D. of 3 independent experiments. **P < 0.01; ***P < 0.001 respect to control cells for each time, using t-tests with Holm-Šídák method for multiple comparisons

To assess activation of a DNA damage response signaling pathway mediated by P53 in HCT-116 cells, phosphorylated p53 (phospho-p53, Ser15) and total P53 levels were analyzed by indirect immunofluorescence at 0.5, 3, 6, and 24 h after exposure to 10 radiation pulses (Fig. 6). A marked and statistically significant increase in phospho-p53 fluorescence intensity was observed as early as 0.5 h post-irradiation and remained elevated up to 24 h, compared with control cells (**p < 0.001, Fig. 6A and B). Although total P53 levels remain constant until 24 h post irradiation (Fig. 6C and D), these results indicate a rapid and sustained activation of p53 signaling in response to radiation-induced DNA damage.

**Fig. 7:**
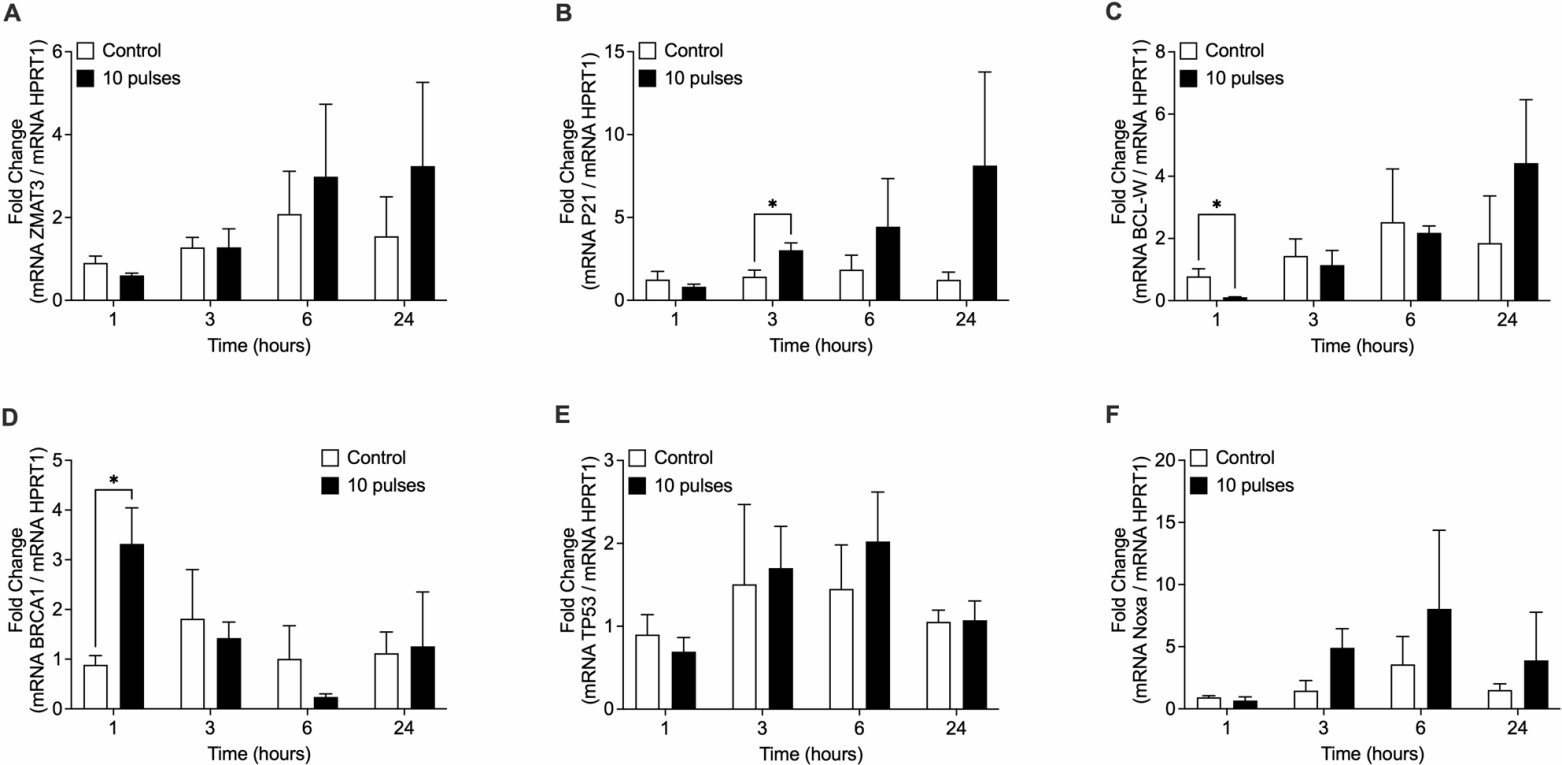
Expression of DNA damage response-associated genes. Expressions levels of **A** ZMAT3, **B** P21, **C** BLC-w, **D** BRCA1, **E** TP53, and **F** NOXA transcripts were evaluated by RT-qPCR in HCT-116 cells at 1, 3, 6 and 24 h after exposition with 0 (control) or 10 pulses of radiation. Expression levels were normalized to HPRT1 housekeeping gene. Means ± S.D of 3 independent experiments are shown. Results are presented relative to control of each time. *P < 0.05, using t-test with Holm-Šídák method for multiple comparisons

Nevertheless, expression of P53 target genes *ZMAT3* and *PMAIP1/NOXA*, exhibited a sustained but not significant increase over 1, 3, 6, and 24 h after exposure to 10 radiation pulses, while *CDKN1A* (p21) showed a significant upregulation at 3 h post-irradiation (Fig. 7). On the other hand, the expression of other genes involved in the DNA damage response, BRCA1 (DNA repair) and BCL2L2/BCL-W (apoptosis), showed a significant increase and decrease 1 h after irradiation, respectively. No significant differences were detected after that time point (Fig. 7C, D). *TP53* showed no significative changes.

Together, these data suggest that pulsed radiation-induced DNA damage activates an early DNA damage response. However, this response would not be sustained in time and does not correlate with the magnitude of γ-H2AX and pP53 signals observed up to 24 h after irradiation nor with the levels of cell death.

**Fig. 8:**
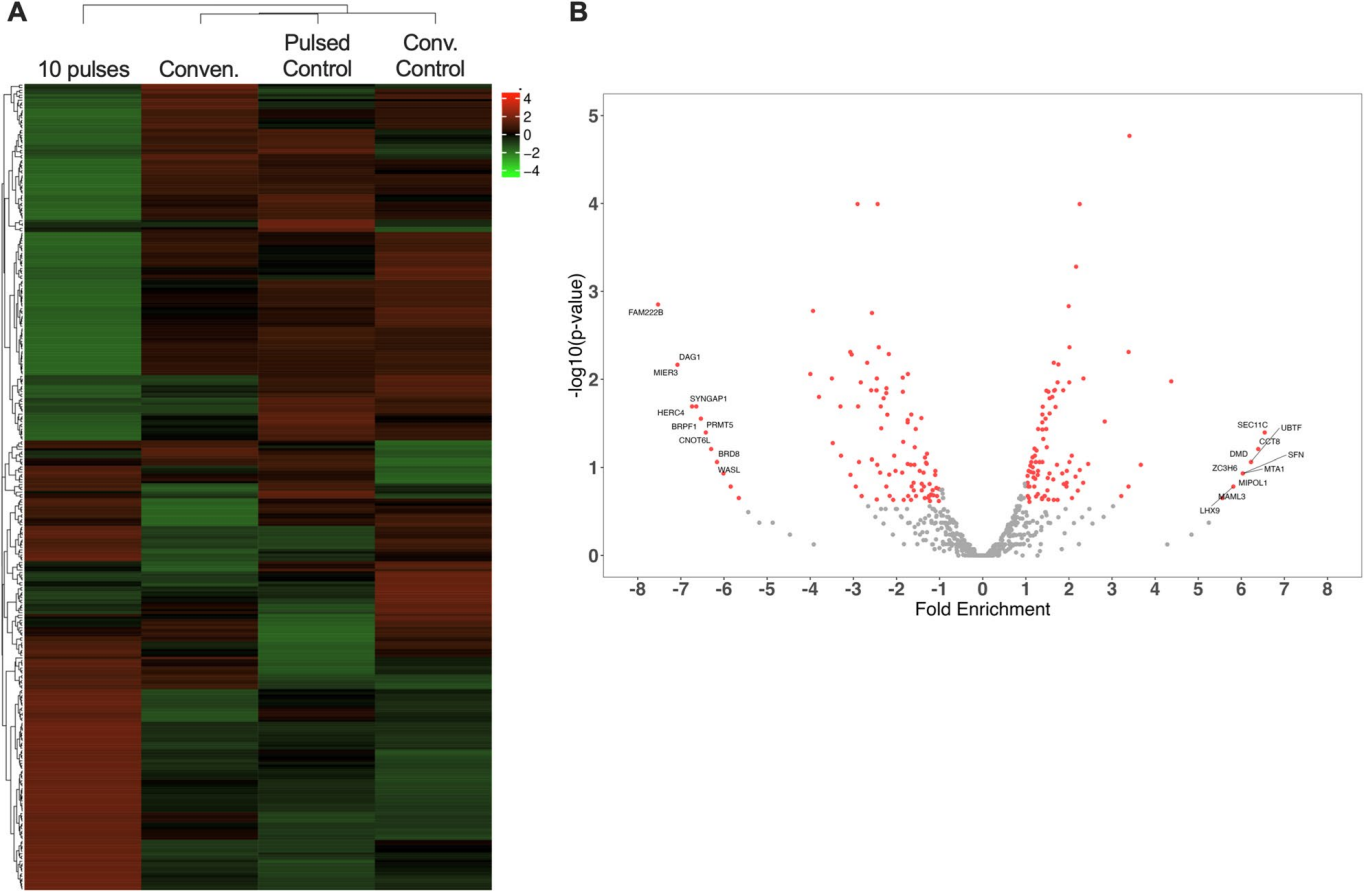
Pulsed radiation-induced differential gene expression: a exploratory study. mRNAs obtained from HCT-116 cells exposed to 10 pulses (0.25 Gy) or nonirradiated were evaluated by RNAseq analyses. Heatmap (**A**) and (**B**) Volcano plot of mRNAs expression profile obtained from RNAseq at 48 h after exposure to Pulsed x-ray to non-irradiated cells (control). Overexpressed mRNAs (Fold Change ≥ 2) and underexpressed (Fold change ≤ 2) with *P* < 0.05 are labeled. Data correspond to a single biological replicate (*n* = 1)

Thus, to elucidate the intriguing pulsed X-rays-induced cellular response leading to cell death, we performed an exploratory transcriptomic analysis in HCT-116 cells at 48 h post-irradiation. Pulsed X-rays induced a notoriously distinct gene expression profile compared to both control and conventional X-ray group (Fig. 8A). A total of 222 genes were differentially expressed, with 105 genes overexpressed and 117 underexpressed (p value < 0.05) (Fig. 8B). However, given the exploratory character of the study (with no biological replicates), only 42 protein coding genes remained significantly dysregulated after multiple testing correction (Benjamini-Hochberg method) (Supplementary Table 1).

**Fig. 9:**
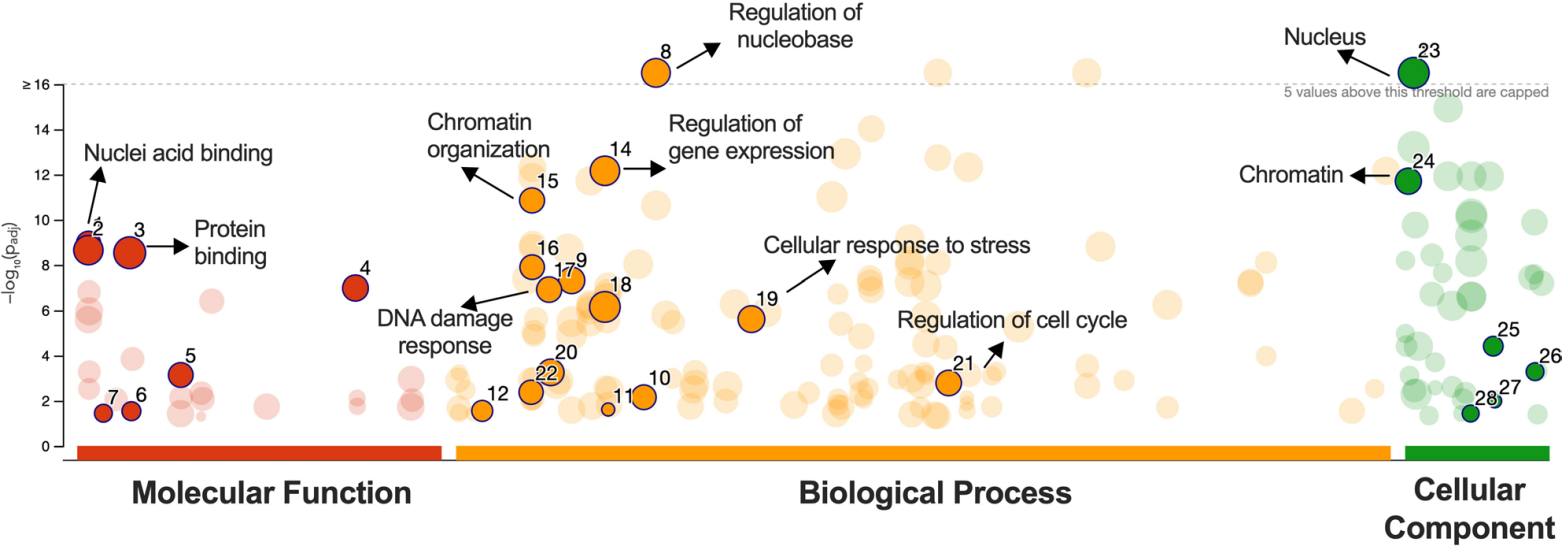
Dysregulated expression induced by pulsed X-rays is associated with cellular response to stress. Enrichment analysis was performance for the most differentially expressed mRNAs, categorized by Molecular function (red columna), Biological process (yellow column) and Cellular component (green column), using g: Profiler data base. Most significant term were labels

Given the limited feasibility of performing meaningful ontological enrichment analyses with this reduced set of genes, ontological analyses were conducted using the initial set of 222 genes, selected based on unadjusted p-values below the significance threshold Fig. 9.

The functional enrichment analysis, using g: Profiler, revealed a significant enrichment of “DNA damage response” (*MBD2*,* BRD1*,* MTF2*,* MSL1*,* BRPF1*,* KAT6B*,* ANP32E*,* H3F3B*,* FBXL19*,* PHF21A*,* CHTOP*,* OGT*,* SUZ12*,* TET3*,* TOP1* and *SPEN*) and “cell cycle regulation” (*BTG1*,* PPM1A*,* YWHAE*,* CCNL1*,* MEIS2*,* EIF4E*,* YTHDF2*,* PIM3*,* HES1*,* PUM2*,* UBA3*, and *SON*) among other biological processes. Interestingly, a significant overrepresentation of genes associated with “chromatin organization” (*MBD2*,* SMARCE1*,* BRD1*,* MTF2*,* MSL1*,* ARID1A*,* PRMT5*,* BRPF1*,* KAT6B*,* DYRK1A*,* ATRX*,* ANP32E*,* H3F3B*,* BRD8*,* SFPQ*,* UBR5*,* WAC*,* CHD2*,* FBXL19*,* ENY2*,* MTA1*,* PHF21A*,* EPC2*,* TAF10*,* BRD4*,* PBRM1*,* CHTOP*,* CBX5*,* OGT*,* KDM2A*,* SUZ12*,* KAT5*,* TET3*,* TOP1*,* SPEN*, and *MORF4L2*). This, together with other dysregulated biological processes like “regulation of nucleobase” and “regulation of gene expression”, suggest a potential chromatin remodeling event facilitating differential gene expression as part of the cellular response to pulsed radiation.

## Discussion

The biological phenomenon known as the FLASH effect is defined by the delivery of radiation at ultra-high dose rates above a prescribed threshold [33]. The consensus threshold for the mean dose rate is generally defined as exceeding 40 Gy/s, with many research groups targeting 100 Gy/s or greater. Crucially, this effect relies on the overall irradiation time being extremely brief, typically less than 500 milliseconds, a timeframe thought necessary to facilitate transient biophysical phenomena, such as oxygen depletion [34]. Current accelerator-based FLASH systems achieve exceptionally high intrapulse dose rates, typically ranging from 10^6^ to 10^7^ Gy/s [34]. Plasma focus device, PF-2 kJ, emits X-ray pulses on the order of nanoseconds with a dose of ~ 0.25 Gy per pulse that yields an ultra-high-dose rate (~ 10^7^ Gy/min) with a total low dose. In a recently published study, in vitro experiments revealed larger cell death at much lower doses of pulsed X-ray irradiation compared to conventional X-ray irradiation on the DLD-1 cell line [20] .In the present study, large DNA damage was evidenced in comparison with conventional X-ray irradiation. Such effects of an ultrahigh-dose-rate with a total low dose are motivating to study the pulsed radiation effects on cancer cells. However, the present study is limited to in vitro cell cultures, and in the future, more sophisticated biological models (spheroids, organoids, in vivo) will be used to study the relevance of the plasma focus devices as a pulsed radiation source to be used in FLASH-RT.

The results obtained in this study reinforce the growing evidence on the differential effects of pulsed radiation on cancer cells compared to conventional radiation. In particular, irradiation with ultra-high dose rate (~ 10^7^ Gy/min) pulsed X-rays induced a significant increase in apoptosis in the colorectal cancer cell lines HCT-116 and DLD-1, as well as in the breast cancer cell line MCF-7, whereas minimal or no effect is observed when using conventional radiation at similar doses [25, 29, 35, 36]. These findings align with the core principle of FLASH radiotherapy (FLASH-RT), where the aim is to achieve a differential effect between tumor and healthy tissue. While our study utilizes extremely high instantaneous dose rate pulsed X-rays (from a plasma focus device PF-2 kJ) and focuses on cancer cell death, the consistency of finding a distinct response compared to conventional radiation is a crucial shared theme with the broader FLASH literature [1, 37].

In a study with conventional X-ray irradiation, DLD-1 cell has showed radio-sensitivity at low doses [29], which could explains the difference in cell death between pulsed and conventional X-ray irradiation, however, the response of HCT-116 cell to pulsed X-ray irradiation suggests a change in sensitivity from radioresistant to radio-sensitive at lower dose. Interestingly, one of the most remarkable aspects of this study is the persistence of DNA damage, measured through γ-H2AX foci, after exposure to pulsed radiation. It was observed that the double-strand breaks (DSBs) generated by pulsed radiation were not fully repaired even after 24 h post-irradiation, suggesting a limited capacity of the repair mechanisms under these conditions, which is consistent with a subsequent apoptotic mechanism of cell death. Some studies on FLASH irradiation report a reduction in DNA damage markers, including γH2AX and 53BP1, with UHDRs compared to conventional dose rates [38, 39]. This “sparing effect” is often linked to the rapid dose delivery leading to reduced indirect effects from radical products or enhanced radical recombination [40, 41]. For example, one study found that while γH2AX foci initially increased, but they typically returned to control levels within 24 h, even if some residual foci persisted at higher doses [42]. Our finding of persistent γH2AX foci at 24 h post-irradiation suggests a distinct DNA damage response under pulsed X-rays. Based on the comparison above, the unique contribution of this study lies in identifying a distinct biological signature induced by UHDR Pulsed X-rays (from a plasma focus device) that appears to be mechanistically separable from the widely reported transient oxygen depletion (TOD) -driven normal tissue sparing of accelerator-based FLASH radiation. The central insight is that pulsed X-ray UHDR may operate via a mechanism driven by spatial ionization density (damage complexity), in contrast to the primary radiochemical (TOD/ROS) mechanism that dominates sparing in particle FLASH. The finding of persistent γ-H2Ax foci suggests the formation of highly clustered, irreparable DNA lesions (Complex DNA Damage).

The increase in p53 phosphorylated at serine 15, a marker of p53 activation and stabilization [49–51], 30 min post-irradiation and persisting up to 24 h, support the presence of DNA damage. Transcripts directly regulated by p53, like P21, increased at 3 h post 10 X-rays pulses irradiation. Previous studies, in different cells lines, have shown a dose-dependent relationship for P21 mRNA levels at doses up to 15 Gy [43–45] with a peak observed 2–4 h after radiation exposure [44, 46, 47] and return to control levels at 24 h [46, 48]. Intriguingly, the sustained phosphorylation of p53 together with the presence of γH2AX foci suggests a DNA damage response that ultimately leads to the induction of cell death with no evidence of prior G2/M arrest. Although previous studies have reported dynamic changes in both total and phosphorylated P53 levels following exposure of high doses of conventional radiation [49, 52], our results did not show significant variation in total P53 levels, possibly due to the approximately 40-fold lower doses used in this study. Together, these data suggest that pulsed radiation-induced DNA damage activates an early DNA damage response. However, this response would not be sustained in time and does not correlate with the magnitude of γ-H2AX and pP53 signals observed up to 24 h after irradiation nor with the levels of cell death.

Even so, transcriptomic data shared in this work must be considered as preliminary, it is worth to highlight the notoriously distinct transcriptomic profile induced in pulsed- irradiated cells, which was not observed with conventional radiation exposure. Altogether, this data suggest that pulsed radiation may trigger distinct biological responses leading to cell death. This finding is consistent with recent studies suggesting that pulsed radiation could overcome radio-resistance in certain tumor types by activating alternative apoptotic pathways [9, 21]. Independent biological replicates are mandatory to identify larger differentially expressing gene sets that shed light about the specific underlying molecular mechanism.

From a clinical perspective, the ability to induce apoptosis more efficiently in cancer cells while minimizing damage to normal tissues represents a significant advancement in radiotherapy. In previous studies with FLASH radiation (ultra-high dose in a short time), a “FLASH effect” has been observed, characterized by relative protection of normal tissues without compromising antitumor efficacy [4, 7, 9, 34, 53]. Similar non-toxic effects of pulsed X-ray irradiation (in the order of nano-seconds) have also been reported on the non-cancerous cell line CCD 841 CoN [20]. Although the present study does not directly address this effect in healthy tissues, the results align with the fundamental principle of FLASH-RT, where the Therapeutic Index is enhanced:

$$\begin{gathered} \:Therapeutic\:Index \hfill \\ \quad \: = \frac{{\:Enhanced\:Apoptosis\:in\:Cancer\:\left( {Pulsed\:X - ray} \right)}}{{Minimal\:Toxicity\:in\:Normal\:Tissue\:\left( {Pulsed\:X - ray} \right)\:}} \hfill \\ \end{gathered}$$

This ratio is highly favorable and supports the potential for pulsed X-ray modalities in treating radioresistant tumors cells like HCT-116 and DLD-1.

Several hypotheses have been presented to explain the effect of pulsed radiation on cancerous cells [2, 14, 20, 54]. In the case of FLASH-RT, hypoxia is considered the main reason for the selective effects on cancer cells vs. healthy cells [15, 16, 55, 56]. In the case of pulsed X-ray irradiation using the plasma focus device, the X-ray energy and duration of X-ray pulses were suggested to have larger effects on cancer cells while comparing with conventional X-ray irradiation effects. Our mechanistic data (persistent DSBs) provide the first strong evidence for a DNA damage complexity mechanism driving tumor killing in UHDR pulsed X-ray irradiation, which differs from the established radiochemical mechanisms in other FLASH modalities.

In conclusion, the findings presented in this work contribute to the growing body of evidence supporting the use of pulsed radiation as an effective strategy to induce apoptosis in cancer cells, with persistent DNA damage and distinctive gene regulation compared to conventional radiation. Additional studies, particularly in 3D and in vivo models, are required to evaluate the clinical impact and safety of this strategy before its potential application in clinical settings.

## Supplementary Information

Below is the link to the electronic supplementary material.

Supplementary Material 1

Supplementary Material 2

## Data Availability

The datasets used and/or analysed during the current study are available from the corresponding author on reasonable request.
