# Supplementary material for: DNA damage and cell death induced by exposure to ultra-high dose rate low-dose pulsed X-rays emitted from a kilojoule plasma focus device
Source: Biol Res. 2026 Mar 10;59:22. doi: 10.1186/s40659-026-00674-1 (PMC13088622; doi:10.1186/s40659-026-00674-1)
Supplement: Supplementary file 2 — Supplementary Material 2 [file 40659_2026_674_MOESM2_ESM.docx]

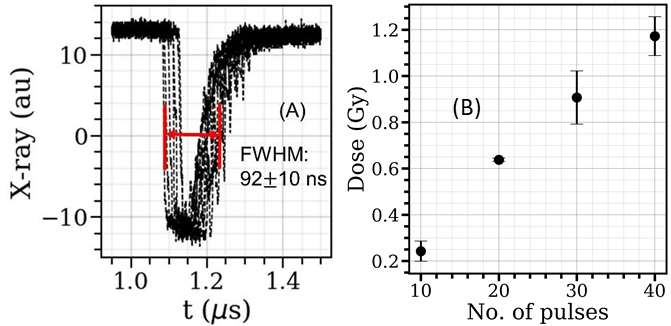


**Figure S1.**

(A) The Full Width at Half Maxima (FWHM) of X-ray pulses (~ 90 ns) captured using a combination of scintillator (Bicron BC-408) and a photomultiplier tube. Adapted from Jain, J., Araya, H., Moreno, J., Davis, S., Andaur, R., Bora, B., ... & Soto, L. (2021). Hyper-radiosensitivity in tumor cells following exposure to low dose pulsed x-rays emitted from a kilojoule plasma focus device. Journal of Applied Physics, 130(16).

(B) Linear relationship between the number of X-ray pulses and dose. Figure 1 (B) shows the variation in doses with the number of X-ray pulses. The standard deviation is due to three independent dose measurements. Besides, doses were monitored before and after cell culture irradiation to ensure the dose repetition for 10 X-ray pulses.
